# Intrinsic anticarcinogenic effects of *Piper sarmentosum *ethanolic extract on a human hepatoma cell line

**DOI:** 10.1186/1475-2867-9-6

**Published:** 2009-03-03

**Authors:** Shahrul Hisham Zainal Ariffin, Wan Haifa Haryani Wan Omar, Zaidah Zainal Ariffin, Muhd Fauzi Safian, Sahidan Senafi, Rohaya Megat Abdul Wahab

**Affiliations:** 1 School of Bioscience and Biotechnology, Faculty of Science and Technology, Universiti Kebangsaan Malaysia, 43600, Selangor Darul Ehsan, Malaysia; 2 Department of Microbiology, Faculty of Applied Science, Universiti Teknologi MARA, 40450, Shah Alam, Selangor, Malaysia; 3 Department of Orthodontic, Faculty of Dentistry, Universiti Kebangsaan Malaysia, 50300, Kuala Lumpur, Malaysia

## Abstract

**Background:**

*Piper sarmentosum*, locally known as kaduk is belonging to the family of Piperaceae. It is our interest to evaluate their effect on human hepatoma cell line (HepG2) for the potential of anticarcinogenic activity.

**Results:**

The anticarcinogenic activity of an ethanolic extract from *Piper sarmentosum *in HepG2 and non-malignant Chang's liver cell lines has been previously determined using (3-[4,5-dimethylthiazol-2-yl]-2,5-diphenyl-tetrazolium bromide) (MTT) assays, where the IC_50 _value was used as a parameter for cytotoxicity. The ethanolic extract that showed anticarcinogenic properties in HepG2 cells had an IC_50 _of 12.5 μg mL^-1^, while IC_50 _values in the non-malignant Chang's liver cell line were greater than 30 μg mL^-1^. Apoptotic morphological changes in HepG2 cells were observed using an inverted microscope and showed chromatin condensation, cell shrinkage and apoptotic bodies following May-Grunwald-Giemsa's staining. The percentage of apoptotic cells in the overall population (apoptotic index) showed a continuously significant increase (p < 0.05) in 12.5 μg mL^-1 ^ethanolic extract-treated cells at 24, 48 and 72 hours compared to controls (untreated cells). Following acridine orange and ethidium bromide staining, treatment with 10, 12 and 14 μg mL^-1 ^of ethanolic extracts caused typical apoptotic morphological changes in HepG2 cells. Molecular analysis of DNA fragmentation was used to examine intrinsic apoptosis induced by the ethanolic extracts. These results showed a typical intrinsic apoptotic characterisation, which included fragmentation of nuclear DNA in ethanolic extract-treated HepG2 cells. However, the non-malignant Chang's liver cell line produced no DNA fragmentation. In addition, the DNA genome was similarly intact for both the untreated non-malignant Chang's liver and HepG2 cell lines.

**Conclusion:**

Therefore, our results suggest that the ethanolic extract from *P. sarmentosum *induced anticarcinogenic activity through an intrinsic apoptosis pathway in HepG2 cells *in vitro*.

## Background

Human hepatocellular carcinoma is the fifth most common cancer in the world and the fourth most common cause of cancer-associated mortality [1]. Surgical resection and local treatment are frequently limited due to metastasis, cirrhosis, and other pathological changes in the liver parenchyma. The synchronous occurrence of human hepatocellular carcinoma may be due to different risk factors such as chronic viral hepatitis B or hepatitis C infection, aflotoxin explosure, alcohol consumption and iron overload [2]. The development of chemotherapeutic or chemopreventive agents for hepatocellular carcinoma is important in order to help reduce the mortality caused by this disease [3]. Thus, significant research efforts have focused on novel chemotherapeutic drugs from the plant kingdom in search of cancer inhibitors and cures [4].

Plants have many phytochemicals with various bioactivities, including antioxidant, anti-inflammatory and anticancer functions. For example, some studies have reported that extracts from natural products such as fruits, vegetables and medicinal herbs have positive effects against cancer compared with chemotherapy or recent hormonal treatments [5]. The family of *Piperaceae *belonging to superorder *Nymphaeifloraea*, order *Piperales *[6], comprises about 10 genera and 2,000 species [7]. The genus *Piper *(Piperaceae) is largely distributed in tropical and subtropical regions of the world. Chemical studies have shown that the genus *Piper *has many components including unsaturated amides, flavonoids, lignans, aristolactams, long and short chain esters, terpenes, steroids, prophenylphenols, and alkaloids [8,9]. Some *Piper *species are used in folk medicine to treat many diseases, including fever, jaundice, rheumatism and neuralgia [7]. In Malaysia, *P. sarmentosum *is locally known as kaduk and is commonly used in folk medicine as a carminative. The leaves and roots of this plant are used for the treatment of toothaches, fungal dermatitis on the feet, asthmatic coughing and pleurisy [10]. In addition, the plant and its fruits are used as an expectorant [11].

Previous studies have investigated other biological activities including the anti-inflammatory effects of *Peperomia pellucida *[12] and the antimicrobial effects of *Piper anducum *[13,14]. Chloroform extracts from *Piper sarmentosum *have also shown considerable antimalarial activity against *Plasmodium falciparum (in vitro*) and *Plasmodium berghei *(*in vivo*) [15]. The water extract of the entire plant showed a hypoglycaemic effect in rats [16], while the methanolic extract from the leaves of *Piper sarmentosum *exhibited peak antioxidant activity [17]. However, the properties of this plant, and especially its anticarcinogenic activity, have not yet been investigated. The objective of this study was to evaluate the anticarcinogenic properties and mode of action of the *Piper sarmentosum *ethanolic extract in a human hepatoma cell line (HepG2). The Chang's liver cell line was used as a non-malignant cell for cytotoxic activity.

## Results and discussion

### Cytotoxic activity of *P. sarmentosum *ethanolic extract on cells

In this study, we investigated the effects of a crude ethanolic extract from *P. sarmentosum *in HepG2 and non-malignant Chang's liver cell lines. In the first part of this study, the antiproliferative properties of the ethanolic extract from *P. sarmentosum *were predetermined using an MTT assay. The principle of this assay is based on the reduction of a soluble tetrazolium salt, by mitochondrial dehydrogenase activity of viable tumour cells, into a soluble coloured formazan product that can be measured spectrophotometrically after dissolution [18]. The IC_50 _value was used as a parameter for cytotoxicity.

**Figure 1:**
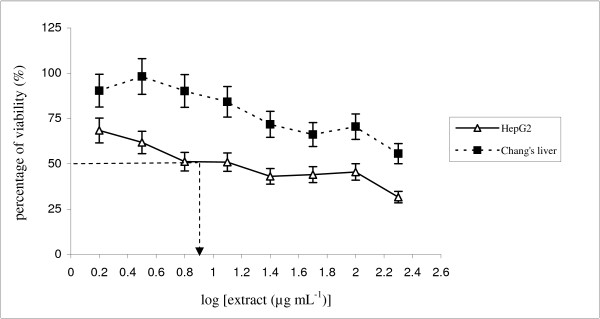
**MTT assaying of the *P. sarmentosum *ethanolic extract in HepG2 and non-malignant Chang's liver cells**. Both cells were treated at various concentrations, i.e., 1.56–200 μg mL^-1^. The IC_50 _value for HepG2 was 12.5 μg mL^-1^, while the IC_50 _value for non-malignant Chang's liver cells was > 30 μg mL^-1^. Each data point represents values from three independent experiments (n = 3).

The criterion for cytotoxicity for the crude extracts, as established by the National Cancer Institute (NCI), is an IC_50 _value lower than 30 μg mL^-1 ^[19]. Figure 1 showed that the *P. sarmentosum *ethanolic extract was able to exert antiproliferative effects in the HepG2 cell line tested in dose-dependent manner. The IC_50 _value of the ethanolic extract for HepG2 cells viability was 12.5 μg mL^-1 ^after exposure for 72 hours (Figure 1). Our results show that the normal counterpart cells (non-malignant Chang's liver) treated with 200 μg mL^-1 ^of ethanolic extract still retained > 50% viable cells, i.e., 55.6% viability. On the other hand, IC_50 _values for the ethanolic extract in non-malignant Chang's liver cells were more than 30 μg mL^-1 ^(Figure 1). Therefore, *P. sarmentosum *ethanolic extract predetermination by MTT assay induced cytotoxicity activity in the hepatoma cell line (HepG2), but not in the non-malignant cell line (Chang's liver).

**Figure 2:**
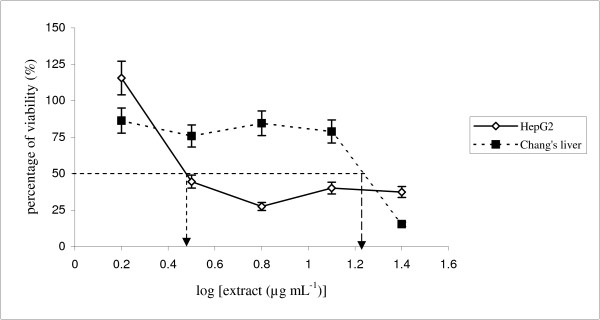
**MTT assaying of tamoxifen in HepG2 and non-malignant Chang's liver cells**. Both cells were treated at various concentrations (1.56–25 μg mL^-1^). The IC_50 _value for HepG2 is 3 μg mL^-1 ^and 18.6 μg mL^-1 ^for non-malignant Chang's liver cells. Each data point represent values from three independent experiments (n = 3).

Comparatively, tamoxifen, a drug with anti-oestrogenic activity, was used in this study as a positive control. Tamoxifen imposed an inhibitory effect in the HepG2 cell line with an IC_50 _value of 3 μg mL^-1 ^and in the non-malignant Chang's liver cell line with a value of 18.6 μg mL^-1^(Figure 2). Therefore, tamoxifen induced cytotoxic activity in both carcinoma (HepG2) and non-carcinoma (non-malignant Chang's liver) cells. Both cells induced IC_50 _below 30 μg mL^-1 ^and were thus considered to induce cytotoxic activity to the treated cells, as recommended by National Cancer Institute (NCI) [19]. NCI recommended that any extract generates IC_50 _below than 30 μg mL^-1 ^is considered possess cytotoxic activity. As a result, MTT assay analysis showed that the ethanolic extract of *P. sarmentosum *induced cytotoxic activity in HepG2 cells, but not in the non-malignant Chang's liver cells. In contrast, an anticarcinogenic drug (tamoxifen) induced cytotoxic activity in hepatocellular carcinoma, HepG2 and non-malignant Chang's liver cell lines.

### Morphological observation

**Figure 3:**
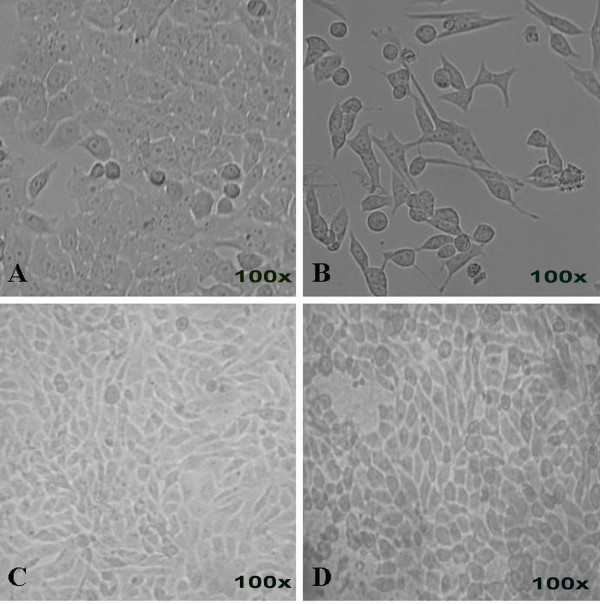
**Morphological studies by inverted microscope at actual magnification 100×**. HepG2 and non-malignant Chang's liver cell line were treated without (A, C) and with 12.5 μg mL^-1 ^of *P. sarmentosum *ethanolic extract (B, D) for 72 hours. Both types of treatment (C and D) produced similar cellular morphology and antiproliferative effect. However, in HepG2 cells, the confluency appeared to be reduced from 90% in untreated cells to 10% in treated cells. Similar cellular morphology was observed in three independent experiments (n = 3).

Light microscopic observation of the *P. sarmentosum *ethanolic extract-treated HepG2 cell line after 72 hours of exposure showed typical morphological features of apoptosis. The characterisation of morphological changes observed were reduction in cell volume, cell shrinkage, reduction in chromatin condensation and formation of cytoplasmic blebs [20]. Figure 3B shows that the HepG2 cells treated with ethanolic extract at 12.5 μg mL^-1^were changed into round shapes as compared to untreated HepG2 cells (Figure 3A). The untreated cells (HepG2) also showed a high confluency of monolayer cells (Figure 3A) compared to ethanolic extract-treated cells, which showed a reduction in cell volume and cell shrinkage (Figure 3B). Figure 3C shows that the morphology of the untreated non-malignant Chang's liver cell line is a confluent monolayer. The non-malignant Chang's liver cell line was then treated with 12.5 μg mL^-1 ^of *P. sarmentosum *ethanolic extract. After 72 hours of incubation, the morphology of the treated non-malignant Chang's liver cell line (Figure 3D) showed similar morphology to that untreated non-malignant Chang's liver cell line (Figure 3C).

### Morphological observation by May-Grunwald Giemsa's staining

**Figure 4:**
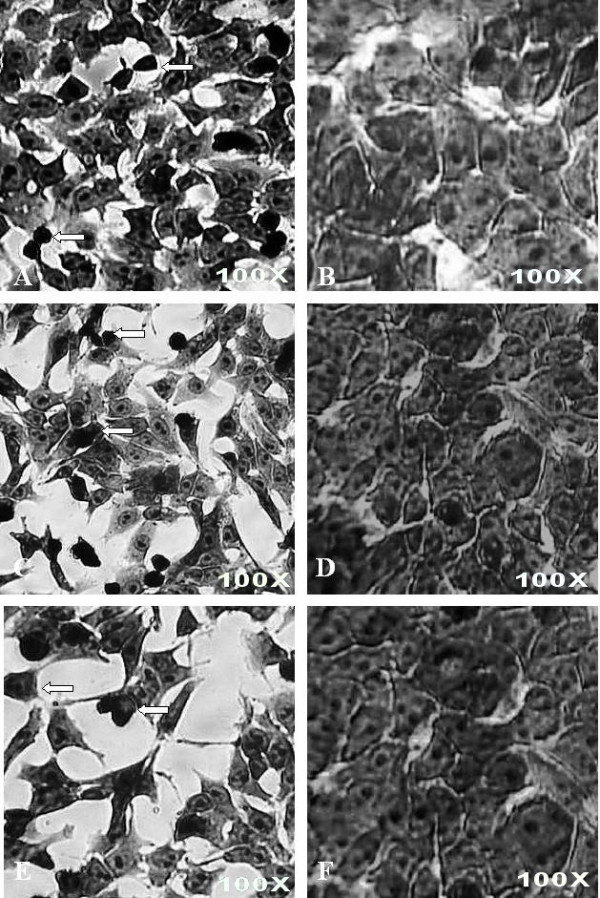
**Morphological observation with May-Grunwald-Giemsa's staining at actual magnification 100×**. HepG2 cells were treated for 24 (A), 48 (C) and 72 (E) hours with 12.5 μg mL^-1 ^of *P. sarmentosum *ethanolic extract while untreated HepG2 cells were grow in complete medium for 24 (B), 48 (D) and 72 (F) hours. The white arrows indicated apoptotic bodies. The figures shown are representative of three independent experiments (n = 3).

Ethanolic extracts from *P. sarmentosum *can induce apoptosis in HepG2 cells, as proven using May-Grunwald-Giemsa's staining (Figure 4). The apoptotic morphological pictures clearly show the appearance of apoptotic bodies (indicated as white arrow) when using an inverted microscope at 100× actual magnification. Marked morphological changes of the apoptotic cells are represented by apoptotic bodies (indicated as white arrow), which are easily determined by May-Grunwald-Giemsa's staining (Figure 4A, C and 4E). These apoptotic cells can be seen when the cells are exposed to 12.5 μg mL^-1 ^of the ethanolic extract for 24, 48 and 72 hours.

### Apoptotic Index (AI)

**Figure 5:**
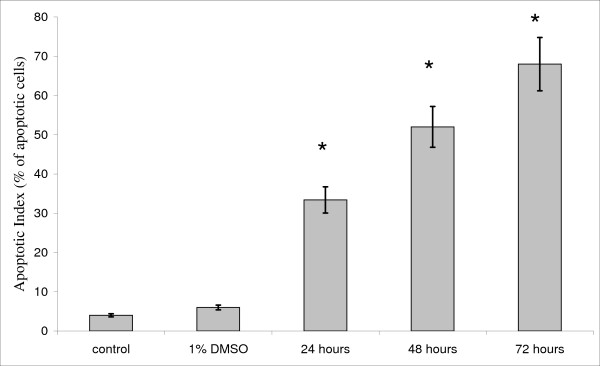
**Percentages of HepG2 cell death via apoptosis after treatment with ethanolic extract from *P. sarmentosum***. The percentages of HepG2 cell death via apoptosis increased significantly in a time-dependent manner. p < 0.05 represents the statistically significant difference between the control and treated group (1% DMSO, 24–72 hours of incubation in 12.5 μg mL^-1 ^of ethanolic extract). *Represents significant results (p < 0.05) using ANOVA statistical analysis when the treated group was compared with the control.

The apoptotic index (AI) was calculated to confirm that ethanolic-treated cell death was via apoptosis. AI is described as the percentage of apoptotic cells and apoptotic bodies within the overall population of cells [21]. An apoptotic index was determined as the percentage of apoptotic cells from at least 400 counted cells under observation using an inverted microscope. The statistical differences between the control group and treated group (1% DMSO, 24 hours, 48 hours and 72 hours) were analysed using ANOVA, and p values less than 0.05 were considered as significant. The percentages of apoptotic cells after treatment were increased in a time-dependent manner with less than 50% at 24 hours, more than 50% at 48 hours and even higher at 72 hours. Untreated cells are represented as the control, i.e., the HepG2 cell line cultured in complete media for 72 hours. The control cells showed that only 4% of these cell deaths produced a typical morphological apoptotic feature (Figure 5). On the other hand, cells treated with 1% DMSO (negative control) for 72 hours produced only 8% cell death and showed no significant difference (p > 0.05) when compared to untreated cells (control). In contrast, Figure 5 also showed that the AI percentage of HepG2 cells increased significantly (p < 0.05) when the HepG2 cell line was treated with 12.5 μg mL^-1 ^of ethanolic extract from *P. sarmentosum *at 24 hours compared to the control. The AI percentage of the HepG2 cell line also continued to increase significantly (p < 0.05) when the HepG2 cell line was treated with ethanolic extracts at 48 and 72 hours compared to the control (Figure 5). This observation indicated that the apoptotic activity was gradually increased when the ethanolic extract was incubated longer in carcinoma HepG2 cells.

### Morphological observation by acridine orange and ethidium bromide (AO/EB) staining

**Figure 6:**
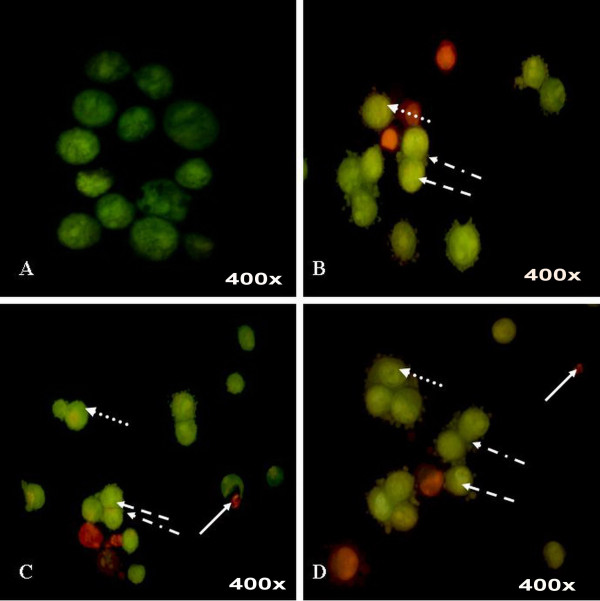
**Morphological observation with acridine orange and ethidium bromide (AO/EB) staining at actual magnification 400×**. HepG2 cells were treated without (A) and with *P. sarmentosum *ethanolic extract, 10 μg mL^-1 ^(B), 12 μg mL^-1 ^(C) and 14 μg mL^-1 ^(D) for 72 hours. Dashed arrow indicated cells with chromatin condensation; rounded dotted arrow indicated cells with fragmented nuclei; dashed dotted arrow indicated cells with membrane blebbing and full white arrow indicated the presence of apoptotic bodies. Each experiment was performed in triplicate (n = 3) and generated similar morphological features.

Staining cells with fluorescent dyes, including acridine orange and ethidium bromide, is used in evaluating the nuclear morphology of apoptotic cells. To corroborate that apoptosis has been induced by *P. sarmentosum *ethanolic plant extract, HepG2 cells were analysed in the presence of acridine orange and ethidium bromide staining (AO/EB staining). Acridine orange is a vital dye that will stain both live and dead cells, whereas ethidium bromide will stain only those cells that have lost their membrane integrity [22]. Three different concentrations were chosen based on the IC_50 _values determined by MTT assay, which were 10, 12 and 14 μg mL^-1^. As a control, HepG2 cells were cultured in complete media and stained with AO/EB (Figure 6A). The figure shows that the ethanolic extract from *P. sarmentosum *induced apoptosis after 72 hours incubation at all concentrations of plant extract tested. Cells stained green represent viable cells, whereas yellow staining represented early apoptotic cells, and reddish or orange staining represents late apoptotic cells. As shown in Figure 6B, HepG2 cells treated with 10 μg mL^-1 ^of ethanolic extract showed changes in cellular morphology, including chromatin condensation, membrane blebbing, and fragmented nuclei. On the other hand, Figures 6C and 6D show similar features for cells treated with 10 μg mL^-1 ^of ethanolic extract (Figure 6B), but with extra features of late stage apoptotic activity with apoptotic bodies when HepG2 cells were treated with 12 μg mL^-1 ^and 14 μg mL^-1 ^of ethanolic extract from *P. sarmentosum*. Therefore, using the AO/EB staining procedure, the morphological features of a hepatoma cell line in apoptosis were dose dependent, i.e., a stronger apoptosis signal was induced with higher concentrations of the respective extract.

### Determination of intrinsic apoptosis by DNA fragmentation

**Figure 7:**
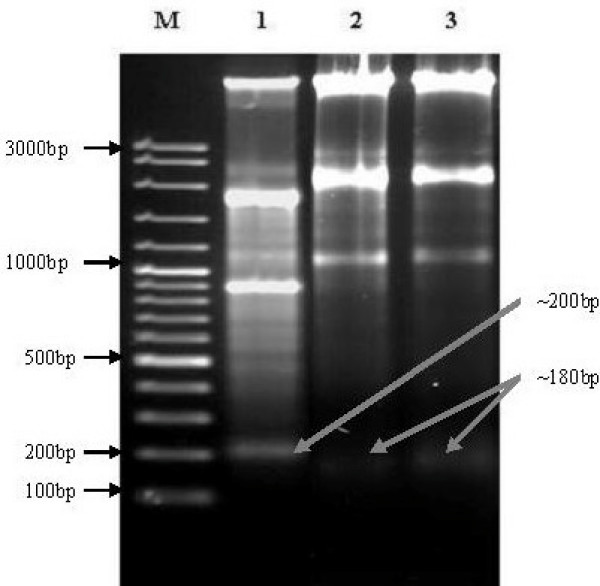
**Gel electrophoresis of DNA genomes extracted from various HepG2 cells following treatment**. Cells were incubated with various concentrations of ethanolic extract for 72 hours. DNA fragments were separated using 1.5% agarose gel electrophoresis and visualised under UV light after staining with ethidium bromide. M: 100 bp DNA ladder marker, lane 1: HepG2 treated with 10 μg mL^-1 ^of *P*. *sarmentosum *ethanolic extract, lane 2: HepG2 treated with 12 μg mL^-1 ^of *P*. *sarmentosum *ethanolic extract and lane 3: HepG2 treated with 14 μg mL^-1 ^of *P*. *sarmentosum *ethanolic extract. Each experiment was performed in triplicate (n = 3).

**Figure 8:**
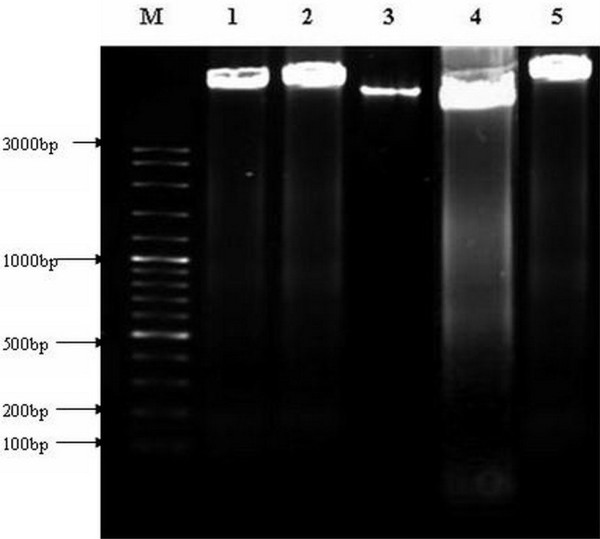
**Gel electrophoresis of DNA genomes extracted from various treated Chang's and untreated HepG2 cells**. Cells were incubated without or with various concentrations of ethanolic extract for 72 hours. The DNA fragments were separated using 1.5% agarose gel electrophoresis and visualised under UV light after staining with ethidium bromide. M: 100 bp DNA ladder marker, lane 1: non-malignant Chang's liver treated with 10 μg mL^-1 ^of *P*. *sarmentosum *ethanolic extract, lane 2: non-malignant Chang's liver treated with 12 μg mL^-1 ^of *P*. *sarmentosum *ethanolic extract, lane 3: non-malignant Chang's liver treated with 14 μg mL^-1 ^of *P*. *sarmentosum *ethanolic extract, lane 4: untreated Chang's liver cells and lane 5: untreated HepG2 cell line. Each experiment was performed in triplicate (n = 3).

DNA fragmentation occurs in cells that produce intrinsic apoptosis activity when induced by a variety of agents. This cleavage produces ladders of DNA fragments that are the size of integer multiples of a nucleosome length (180–200 bp) [23]. The DNA fragmentation is initiated by caspase 3 activation of inactive CAD (caspase activated deoxyribonuclease) through removal of its inhibitors, i.e., ICAD [6]. As a biochemical hallmark of intrinsic apoptotic cell death, DNA fragmentation was used to determine whether the antiproliferative effect of *P. sarmentosum *ethanolic extract on cells acts through the respective apoptosis pathway [24]. As shown in Figure 7, the treatment of HepG2 cells with ethanolic extract resulted in the induction of intrinsic apoptosis activity at concentrations as low as 10 μg mL^-1^. HepG2 cells were treated with three different concentrations of ethanolic extract (10, 12 and 14 μg mL^-1^) based on the IC_50 _that was predetermined by MTT assay. HepG2 cells treated with different concentrations of ethanolic extract (Lane 1–3; Figure 7) for 72 hours showed typical features of DNA laddering on an agarose gel, whereas untreated cells produced intact genomes (Lane 5; Figure 8). In contrast, the non-malignant Chang's liver cell line when treated with the various concentrations of ethanolic extract (Lane 1–3; Figure 8) produced similar genomic DNA features as in untreated non-malignant Chang's liver (Lane 4; Figure 8) and HepG2 (Lane 5; Figure 8) cell lines. Therefore, the ethanolic extract at a concentration as low as 10 μg mL^-1 ^can induce nucleosomal DNA fragmentation of HepG2 due to intrinsic apoptosis processes, but not in the non-malignant Chang's liver cell line. In this study, the reason of using "HepG2" and "Chang" liver cells because HepG2 are the model of hepatocellular carcinoma while Chang liver cells are considered as an *in vitro *model of non-malignant or non-tumor liver cells. This is based on other studies such as Antonin *et al*. and Teck *et al*. stated that Chang as non-malignant cells [25,26].

## Conclusion

*P. sarmentosum *ethanolic extract showed a profound effect on a human hepatoma cell line (HepG2) by exhibiting its cytotoxicity towards this cell, i.e., IC_50 _12.5 μg mL^-1^. In contrast, the respective extract did not induced a cytotoxic effect in a non-malignant cell line (Chang's liver cell line), i.e., IC_50 _> 30 μg mL^-1^. Moreover, this ethanolic extract, through morphological analysis using May-Grunwald-Giemsa's and AO/EB staining procedures, showed that the extract was able to trigger cell death through apoptosis. Our observation using AO/EB staining also showed that the extract was able to induce apoptotic activity at concentrations as low as 10 μg mL^-1^. In addition, molecular analysis through DNA fragmentation revealed that the cell death mode of action occurred by an intrinsic apoptosis pathway.

## Methods

### Plant Materials

Fresh plant materials were collected from the Forest Research Institute of Malaysia (FRIM), Kuala Lumpur, Malaysia. Plant extraction was conducted at the Microbiology Laboratory, Faculty of Applied Science, Universiti Teknologi MARA (UiTM) from July 2006 to December 2006. The anticarcinogenic activity of HepG2 and non-malignant Chang's liver cell lines was conducted at Laboratory 4172 and 4176, School of Bioscience and Biotechnology, Faculty of Science and Technology, Universiti Kebangsaan Malaysia (UKM) from January 2007 to October 2008.

### Preparation of plant materials

Plant materials were dried in an oven at 50°C and then soaked three times in ethanol. The extracts were then filtered and concentrated under reduced pressure using a rotary evaporator (Buchi V-500) at 40°C. The concentrated ethanolic extract was dissolved in 1% DMSO to generate various concentrations of extract (1.56–200 μg mL^-1^) for further analyses.

### Cell culture

The human hepatoma (HepG2) and non-malignant Chang's liver cell lines were kindly provided by Dr. Fadilah Rajab of the Universiti Kebangsaan Malaysia (UKM). Both cells were cultured in RPMI 1640 (Flowlab) supplemented with 10% foetal bovine serum (FBS; Gibco), penicillin (50 U mL^-1^) and streptomycin (50 μg mL^-1^) (Gibco). Cells were maintained in humidified air with 5% CO_2 _at 37°C. Cells were harvested using 0.25% trypsin (Hyclone) when they were 70–80% confluent in culture.

### MTT assay

Briefly, 200 μL of cells (1 × 10^4 ^cells) were seeded into 96-well plates and incubated overnight. The following day, cells were then treated with 20 μL of various concentrations of extract (1.56–200 μg mL^-1^) and tamoxifen (positive control) before further incubation for 72 hours. At the end of this incubation, 20 μL of MTT (Sigma) (2 mg mL^-1 ^in PBS) was added to each well and incubated for another 4 hours at 37°C. The formazan crystals were dissolved in 100 μL dimethylsulphoxide (DMSO) and the absorbance was determined at 540 nm using a multi-plate reader (BIO-RAD model 680). The absorbance value that was determined for cells cultured in complete media without plant extract was based on 100% viable cells. Each concentration of the extract was assayed in triplicate.

### Cell observation using an inverted microscope

HepG2 cell lines were grown in 6-well plates and treated with *P. sarmentosum *ethanolic extract. The cells were then washed with 1× Phosphate Buffer Saline (PBS) (Sigma). Morphological and confluency changes in the cells in both the treated group (12.5 μg mL^-1 ^of ethanolic treated-cells incubated for 24, 48 and 72 hours and 1% DMSO treated-cells for 72 hours) and untreated group were observed using an inverted microscope (Nikon TMS).

### Apoptosis analysis

#### Giemsa staining

Briefly, the HepG2 cell line was seeded at 1 × 10^5 ^cells/well in 6-well plates (BD Labware, England), and the plates were then incubated overnight at 37°C. After incubation, ethanolic extracts of various concentrations (10, 12 and 14 μg mL^-1^) were added and incubated for an additional 24 hours. The plates were washed with 1× Phosphate Buffer Saline (PBS), and the cells were stained with May-Grundwald (BDH Chemical Ltd) for 4 minutes. The slides were then rinsed with sterile water and flooded with freshly prepared Giemsa's stain solution (BDH Chemical Ltd) for 6 minutes. Dyestuff was discarded and rinsed again three times with sterile water. Morphological changes were examined using an inverted microscopy (Nikon, TMS) with 100× actual magnification.

#### Acridine orange and ethidium bromide staining (AO/EB staining)

For this purpose, cells were seeded in 6-well plates for 24 hours and then treated with different concentration ranges (10, 12 and 14 μg mL^-1^) for 72 hours. After harvesting by trypsinisation, cells were washed with 1× PBS once. Twenty-five microlitres of the cell suspension was then mixed with 1 μL of the dye mixture, containing 100 mg mL^-1 ^of acridine orange (Sigma) and 100 mg mL^-1 ^of ethidium bromide (Sigma) in 1× PBS. After staining, cells were visualised immediately under a fluorescence microscope (Leica DM 2500).

#### Intrinsic apoptosis as determined by DNA fragmentation

Cells were lysed with lysis buffer (10 mM Tris-HCL, 5 mM EDTA, 200 mM NaCl, 0.2% SDS) and incubated at 60°C for 5 minutes. The sample was digested with 2.5 μL of proteinase K (more than 3 U μL^-1^) (Sigma) and 5 μL of RNase A (1 U μL^-1^) (Fermentas) and was further incubated at 60°C for 1 hour. After this, 250 μL of 5 M NaCl was added and mixed and then incubated on ice for 5 minutes to precipitate proteins. Cells were then centrifuged for 15 minutes at 10,000 rpm and the supernatant was transferred to a fresh tube, to which an equal volume of isopropanol was added to precipitate the DNA, and the sample was centrifuged for 10 minutes at 10,000 rpm. The supernatants were then discarded, and the pellets were washed with 70% cold ethanol. DNA samples were electrophoresed on a 1.5% agarose gel for 1 hour and 30 minutes at 70 V. Finally, the gel was examined under UV light following ethidium bromide staining to determine apoptotic DNA fragmentation.

#### Data analysis

The analysis of variance (ANOVA) was used to determine differences between treated and control groups using Microsoft™ Excel 2007 software. p values less than 0.05 (p < 0.05) were considered statistically significant.

## Abbreviations

HepG2: Human hepatoma cell line; MTT: (3-(4,5-dimethylthiazol-2-yl)-2,5-diphenyl-tetrazolium bromide); AI: apoptotic index; CO_2_: carbon dioxide; DMSO: dimethylsulfoxide; ANOVA: analysis of variance; AO/EB: acridine orange and ethidium bromide; CAD: caspase activated deoxyribonuclease; ICAD: inhibitor caspase activated deoxyribonuclease; UV: ultraviolet; IC_50_: inhibition concentration to kill 50% of cells population; RPMI: Roswell Park Memorial Institute; FBS: foetal bovine serum; PBS: Phosphate Buffered Saline; HCl: hydrochloride acid; EDTA: ethylenediaminetetraacetic acid disodium salt dehydrate; NaCl: natrium chloride; DNA: deoxyribonucleic acid; NCI: National Cancer Institute.

## Competing interests

Before this, there are other researchers working on this *P. sarmentosum *extract. However they are working towards antimalarial, antioxidant and hypoglycaemic effect.

## Authors' contributions

SHZA involved in scientific approach on cell cultures analysis and molecular analysis. He is the head of this particular project and supervisor of WHHWO. Therefore, he acts as the first corresponding author of this manuscript. RMAW and SS are research associate who was involved in cell culture analysis, molecular analysis (advised by SS) and also clinical potential of the generated extract (advised by RMAW as clinician). WHHWO is the postgraduate student that doing the laboratory work and generating all the results. ZZA involvement as the head researcher on scientific approached in determine the plant to be analyses and extraction procedures of the plant. Her research associate is MFS. Therefore her involvements warrant her to become second corresponding author of this manuscript on the plant extraction approached.
